# Supplementary material for: Blanding’s turtles (Emydoidea blandingii) as a reservoir for Leptospira spp
Source: PLoS One. 2019 Jun 6;14(6):e0210688. doi: 10.1371/journal.pone.0210688 (PMC6553713; doi:10.1371/journal.pone.0210688)
Supplement: S1 Table — Results of sampling 34 Blanding’s turtles, including sex, age category, location within DuPage County Forest Preserve, Leptospira spp. PCR results from urine and cloacal swabbing, serovars detected through MAT, and highest titer present. Sex: F = female, M = male, UNK = unknown; Immunoreactive serovars are as listed: A = Autumnalis, B = Bratislava, C = Canicola, I = Icterohaemorrhagiae, H = Hardjo, P = Pomona. (DOCX) [file pone.0210688.s001.docx]

| **Turtle no.** | **Sex** | **Age** | **Location** | **PCR +/-** | **Immunoreactive serovar(s)** | **Highest titer** |
| --- | --- | --- | --- | --- | --- | --- |
| 1 | F | Adult | 1 | - | H | 1:200 |
| 2 | F | Adult | 5 | - | A, I | 1:100 |
| 3 | F | Adult | 2 | + | A, B, P | 1:200 |
| 4 | F | Adult | 5 | + | A | 1:200 |
| 5 | F | Adult | 2 | - | A | 1:200 |
| 6 | F | Adult | 2 | + | A | 1:200 |
| 7 | F | Adult | 2 | + | A | 1:400 |
| 8 | F | Adult | 2 | + | H | 1:200 |
| 9 | M | Adult | 5 | + | A | 1:100 |
| 10 | M | Adult | 5 | + | H | 1:100 |
| 11 | F | Adult | 3 | - | H | 1:800 |
| 12 | F | Adult | 2 | + | H | 1:100 |
| 13 | M | Adult | 4 | + | I | 1:100 |
| 14 | M | Adult | 4 | + | A, C, H, I, P | 1:50 |
| 15 | M | Adult | 4 | + | A, C, H, I | 1:50 |
| 16 | F | Subadult | 5 | + | A | 1:400 |
| 17 | F | Adult | 1 | - | A, I | 1:200 |
| 18 | F | Adult | 2 | + | H, I | 1:50 |
| 19 | F | Adult | 2 | + | A, I | 1:100 |
| 20 | F | Adult | 2 | - | A, H | 1:100 |
| 21 | F | Adult | 2 | - | A | 1:800 |
| 22 | F | Adult | 2 | - | A, H | 1:50 |
| 23 | F | Adult | 2 | + | H | 1:100 |
| 24 | F | Adult | 2 | + | H | 1:400 |
| 25 | F | Adult | 2 | - | H | 1:200 |
| 26 | F | Adult | 2 | + | H | 1:400 |
| 27 | F | Adult | 4 | + | H | 1:100 |
| 28 | F | Adult | 1 | + | H | 1:100 |
| 29 | F | Adult | 4 | + | H, I | 1:200 |
| 30 | M | Adult | 4 | + | H | 1:400 |
| 31 | F | Adult | 4 | + | A | 1:100 |
| 32 | F | Adult | 4 | + | H | 1:100 |
| 33 | M | Subadult | 2 | + | A, H, I | 1:50 |
| 34 | UNK | Juvenile | 2 | + | A | 1:200 |

**S1 Table**. **Serology and RT-PCR results for Blanding’s turtles from DuPage County Forest Preserve.**  Results of sampling 34 Blanding’s turtles, including sex, age category, location within DuPage County Forest Preserve, *Leptospira* spp. PCR results from urine and cloacal swabbing, serovars detected through MAT, and highest titer present. Sex: F=female, M=male, UNK=unknown; Immunoreactive serovars are as listed: A = Autumnalis, B = Bratislava, C = Canicola, I = Icterohaemorrhagiae, H = Hardjo, P = Pomona.
